# Knowledge and attitudes as correlates of dietary supplement use among adults in Saudi Arabia: a cross-sectional online survey of a predominantly Riyadh-based convenience sample

**DOI:** 10.3389/fpubh.2026.1929339

**Published:** 2026-09-29

**Authors:** Nawaf W. Alruwaili, Abdullah Alsenaidy, Mohammed Suhluli, Hatem Alahmari, Nora Alafif, Aljazi Bin Zarah, May A. Alshahwan

**Affiliations:** Department of Community Health Sciences, College of Applied Medical Sciences, King Saud University, Riyadh, Saudi Arabia

**Keywords:** attitudes, convenience sample, cross-sectional studies, dietary supplements, knowledge, practices, Saudi Arabia, self-rated knowledge

## Abstract

**Background:**

Dietary supplement (DS) use is widespread in Saudi Arabia, but high prevalence does not by itself indicate informed use.

**Objective:**

To assess knowledge, attitudes, and practices (KAP) regarding dietary supplements among adults residing in Saudi Arabia and to identify factors independently associated with use.

**Methods:**

A structured, self-administered Arabic questionnaire adapted from published KAP instruments was distributed online by non-probability convenience sampling to adults (≥18 years) residing in Saudi Arabia between 14 March and 14 June 2026; the achieved sample was predominantly young, male, and Riyadh-based. Current DS use, the dependent variable, was defined as agreement (“agree” or “strongly agree”) with the item “Do you currently use dietary supplements?” and coded 1 = current user, 0 = non-user. Associations were examined using chi-square tests and multivariable binary logistic regression, expressed as adjusted odds ratios (aOR) with 95% confidence intervals (CI).

**Results:**

Of 725 respondents, 291 (40.1%) were current users. Although 97.4% had heard of DS, only 32.0% agreed that they knew the correct doses; the internet was the leading information source (57.0%), whereas pharmacists were named by 3.9%. Attitudes were favorable (82.9% agreed DS are safe when used correctly). Among users, daily use (69.4%) and vitamins (83.5%) predominated, and 56.4% had consulted a health professional. In the multivariable model (*n* = 666; 270 users), following a special diet was associated with higher odds of use (aOR 2.44, 95% CI 1.55–3.85). Relative to the reference categories of excellent self-rated knowledge and positive attitude, moderate (aOR 0.34, 0.22–0.53) and poor self-rated knowledge (aOR 0.11, 0.06–0.20) and neutral (aOR 0.40, 0.27–0.59) and negative attitudes (aOR 0.07, 0.03–0.20) were associated with lower odds; equivalently, users rated their own knowledge more highly and held more favorable attitudes than non-users.

**Conclusion:**

In this convenience sample, DS use was common and attitudinally endorsed, yet self-rated dosing competence was limited, and the routine information channel was dominated by online sources, even though 56.4% of users consulted a professional before starting. Knowledge and attitudes were associated with use, but the cross-sectional design cannot establish the direction of these associations.

## 1 Introduction

Dietary supplements (DS), defined as orally consumed preparations containing vitamins, minerals, amino acids, herbs, or other dietary ingredients intended to complement the diet, have become a routine feature of contemporary health behavior (1, 2). In the United States, more than half of adults report supplement use, and the prevalence has risen over the past two decades (3–6), while global consumption surged during the COVID-19 pandemic as consumers sought immune support (7). The Saudi market has expanded in parallel with rising health awareness and broader Gulf consumption trends (1, 8, 9). This growth is clinically relevant in Saudi Arabia, where micronutrient deficiencies, particularly of iron and vitamin D, remain common across population groups, including women and students, and provide a plausible rationale for supplement use (10–13).

High prevalence, however, does not guarantee informed use. Across Saudi and regional studies, awareness of DS is high, yet practical knowledge of correct dosing and safety is frequently limited, and information is often drawn from unverified online sources rather than from health professionals (8, 14–18). Inappropriate or unsupervised use carries real risks, including vitamin toxicity, adverse effects, and clinically important drug–supplement interactions that a substantial proportion of users do not disclose to their clinicians (19–21), and supplement-related hepatotoxicity is an increasingly recognized cause of liver injury (22). These risks are compounded by uneven regulation and quality control (23, 24). Concerns are amplified among athletes, in whom performance pressure and reliance on non-specialist advice can drive poorly monitored use, and who face additional anti-doping risks from products contaminated with prohibited substances (25–28).

The knowledge–attitude–practice (KAP) framework is well-suited to this problem because it captures not only what people know but how they act, revealing gaps between awareness, perception, and behavior that are central to designing effective interventions (8, 14). Although several Saudi and regional KAP studies exist, most have focused on specific subgroups or single regions, and few have simultaneously examined a general adult sample, a self-identified athlete subgroup, and the correlates of use within the same dataset (8, 14, 29–31).

Against this background, the present study moves beyond describing prevalence to examine the knowledge- and attitude-related correlates of DS use. Two recent Saudi surveys frame the current evidence. Alshehri et al. (8) reported KAP profiles in Western Saudi Arabia using descriptive frequencies, without modeling the association between KAP level and use; Al Turki et al. (31) modeled determinants of use in a national sample but characterized them mainly in demographic terms (sex, education, income) and did not include scored knowledge or attitude categories as explanatory variables. Neither study characterized a self-identified athlete subgroup within the same general-adult sample. The present study advances beyond this work in three specific respects: it enters scored, categorized self-rated knowledge and attitude levels into a single multivariable model alongside the full sociodemographic and lifestyle set, so that their associations are estimated mutually adjusted rather than in isolation; it reports the model with formal discrimination and calibration diagnostics, including a calibration plot, rather than point estimates alone; and it profiles sports-supplement and anti-doping practices in an athlete subgroup drawn from the same respondents. We therefore (i) characterized knowledge, attitudes, and practices and the dissociation between awareness and self-rated practical competence; (ii) identified the sociodemographic, lifestyle, and KAP factors independently associated with current use using multivariable logistic regression; and (iii) described sports-supplement practices, including anti-doping behaviors, among self-identified athletes. Because the design is cross-sectional and the sample was recruited by convenience, all associations are reported as correlates rather than as causes or determinants of use, and the findings are intended to generate hypotheses for pharmacist- and dietitian-led counseling and digital health-information strategies rather than to be generalized to the Saudi adult population.

## 2 Materials and methods

### 2.1 Study design, setting, and participants

This cross-sectional study used a structured, self-administered questionnaire delivered online via Google Forms to adults residing in Saudi Arabia between 14 March and 14 June 2026. Reporting follows the Strengthening the Reporting of Observational Studies in Epidemiology (STROBE) statement for cross-sectional studies (32); the completed checklist is provided as a submission file. Participants were eligible if they were aged 18 years or older, resided in Saudi Arabia, and provided electronic informed consent; those who declined consent, were younger than 18 years, or resided outside Saudi Arabia were excluded.

Recruitment used non-probability convenience sampling. The survey link, accompanied by a short plain-language description of the study, was distributed through two channels only: it was posted publicly on the investigators’ X (formerly Twitter) accounts, and it was circulated through WhatsApp to personal and professional contacts and groups. Recipients were encouraged to forward the link to eligible contacts, so that propagation within WhatsApp was partly of a snowball type. No other platform, mailing list, or recruitment route was used. No gym, fitness center, university, sports club, or other institution was enrolled as a formal recruitment partner, and no incentives were offered for participation. Because parts of the investigators’ networks are connected to sport and fitness communities, the link is likely to have propagated preferentially within those communities; we regard this as the most plausible explanation for the high proportion of self-identified athletes in the achieved sample (48.0%) and address it explicitly as a limitation.

The link was shared publicly rather than sent to an enumerated sampling frame, and Google Forms does not record the number of link views or link openings. A response rate therefore cannot be calculated, and the analyzed responses should be understood as an achieved rather than a sampled denominator. Eligibility criteria (age ≥ 18 years, residence in Saudi Arabia) and consent were self-declared: the consent item was presented first and functioned as a gate to the remainder of the form, and age band and region of residence were captured as categorical items. Because the survey was anonymous and required no sign-in, independent verification of age, residence, or identity was not possible; this is acknowledged as a limitation.

For the same reason, technical prevention of repeat submission was not available: no sign-in was required, no personal identifiers or IP addresses were collected, and Google Forms was not configured to restrict responses to one per account. Duplicate submissions were therefore screened for *post hoc* by identifying records with identical values across all 40 substantive questionnaire items (Q2–Q41); two such records were identified and the later submission of each pair was removed. All questionnaire items other than consent were optional, so some respondents elected not to disclose particular fields. Records from respondents who declined consent were removed before analysis; among consenting respondents, every record contained a valid response to the outcome item. Records lacking one or more key sociodemographic responses (age, sex, marital status, education, or region of residence) were also excluded, leaving 725 respondents for the descriptive analysis. Percentages for the remaining variables are reported on variable-specific (available-case) denominators, and the multivariable model was restricted to respondents with complete data on every covariate. The participant flow is summarized in Figure 1.

**Figure 1:**
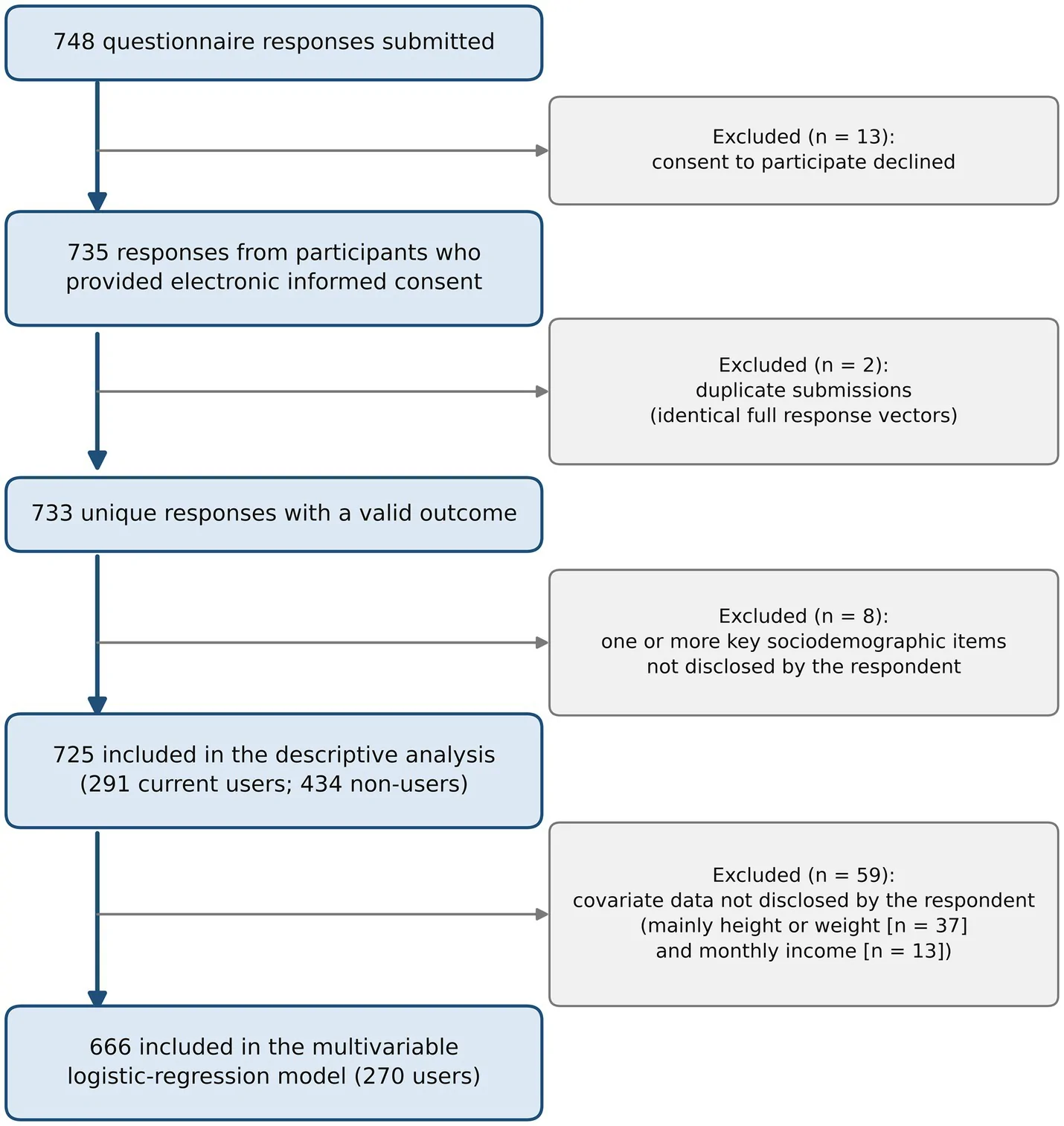
Flow of respondents through the study, from questionnaire submission to inclusion in the descriptive and multivariable analyses. Non-probability convenience sample of adults residing in Saudi Arabia, recruited online between 14 March and 14 June 2026. Boxes on the left show the number of records retained at each stage; boxes on the right show the number excluded and the reason for exclusion. Duplicate submissions were identified *post hoc* as records with identical values across all 40 substantive questionnaire items (Q2–Q41). Descriptive analyses used the 725 respondents retained after these exclusions, with variable-specific (available-case) denominators for items that some respondents chose not to disclose; the multivariable binary logistic regression model was restricted to the 666 respondents (91.9%) with complete data on every covariate.

### 2.2 Questionnaire, translation, and reliability

The questionnaire was developed in Arabic from previously published KAP instruments on DS use and adapted to the study objectives. The general KAP items were adapted from the Arabic questionnaire used by Alshehri et al. (8), and the athlete module from Sadek et al. (25). It comprised five sections: (i) sociodemographic and lifestyle characteristics (age, sex, marital status, education, region, employment, income, chronic disease, smoking, special diet, height, and weight); (ii) knowledge (awareness, self-rated knowledge, self-rated knowledge of recommended doses, awareness of risks, and main information source); (iii) attitudes (perceived benefit, safety, necessity, and willingness to recommend; the willingness-to-recommend item was presented as Q29, after the practice items, in the online form, but was scored together with the other three attitude items); (iv) practices (frequency, type, duration, professional consultation, reasons, and adverse effects); and (v) a conditional athlete module (sports-supplement types, goals, label reading, guidance source, anti-doping checks, and purchasing source) administered only to participants who self-identified as athletes engaged in regular training or competition. The knowledge, attitude, and practice items were administered to all participants; the practice items were summarized among current users only. The main information source item (Q18) and the guidance source item (Q36) each permitted a single response, whereas supplement type (Q24), reason for use (Q27), sports-supplement type (Q32), and goal of sports-supplement use (Q33) permitted multiple responses. Chronic disease (Q9) and special diet (Q11) were each asked as a single yes/no item, without recording the specific condition(s) or diet type(s); these categories therefore cannot be disaggregated. The sex item (Q3) was phrased in Arabic and translates as “Sex,” with the response options male and female; it is reported throughout as self-reported sex, and gender identity was not asked separately. The full instrument, in Arabic with an English translation, is provided as Supplementary Data Sheet 1.

Items drawn from English-language sources were translated into Arabic and independently back-translated by three bilingual reviewers, with discrepancies reconciled by the research team. Content and face validity were assessed by an expert panel in pharmacy and nutrition, who reviewed each item for relevance and clarity; items were revised by consensus. The questionnaire was pilot-tested with 15 eligible adults (excluded from the analysis) to confirm comprehensibility and completion time.

Internal consistency was examined using Cronbach’s alpha. The attitude scale showed good internal consistency (α = 0.81; four items) and the combined seven-item knowledge-and-attitude scale was acceptable (α = 0.76), whereas the three-item knowledge scale was lower (α = 0.60), as was the four-item athlete sports-supplement subscale (α = 0.61). We retained the three-item knowledge scale for four reasons, while explicitly moderating the strength of the inferences drawn from it. First, alpha is a lower-bound estimate of reliability that is strongly and mechanically dependent on the number of items, so short scales are systematically penalized (33). Second, the mean inter-item correlation, a length-independent index of internal coherence, was 0.35, within the 0.15–0.50 range recommended for a scale intended to sample a moderately broad construct (34), and the corrected item–total correlations were 0.51 (self-rated knowledge), 0.51 (knowledge of doses), and 0.25 (awareness of risks). Third, the lower alpha is attributable mainly to the risk-awareness item, which is conceptually adjacent to but distinct from self-rated competence; the two competence items alone yielded α = 0.74. Fourth, and most importantly, the substantive conclusions do not depend on the scaled variable: item-level distributions are reported in full (Supplementary Tables S1, S2), and a pre-specified sensitivity analysis using the continuous knowledge score in place of the categorized variable reproduced the same associations (Supplementary Table S3). We did not conduct formal construct (factor-analytic) or test–retest validation, so the adapted instrument should be regarded as reliability-screened rather than fully validated, and knowledge-scale associations should be interpreted with corresponding caution.

### 2.3 Sample size

Using Cochran’s formula with a 95% confidence level, an expected proportion of 50%, and a 5% margin of error, the minimum required sample was 384; the achieved sample of 725 exceeded this. We note that this calculation establishes precision for a single proportion under simple random sampling and does not confer representativeness on a non-probability sample.

### 2.4 Variables, outcome definition, and scoring

Outcome (dependent variable). Current DS use was ascertained with a single item (Q22), the English translation of which is “Do you currently use dietary supplements?,” answered on a five-point Likert scale (strongly agree, agree, sometimes agree, disagree, strongly disagree). The item refers to use at the time of completion; no fixed retrospective recall window (for example, the preceding 30 days or 12 months) was specified. For analysis, respondents answering “agree” or “strongly agree” were classified as current users and coded 1; all remaining respondents, including the 150 (20.7%) who answered “sometimes agree,” were coded 0. The supplement categories enumerated in the questionnaire were vitamins, minerals, protein products, and herbal products (Q24), with sports-specific products (protein, creatine, branched-chain amino acids, sports drinks, and electrolytes) enumerated separately in the athlete module (Q32). The instrument did not distinguish supplements taken on prescription or on medical advice from self-initiated use, and did not ask separately about caffeine or energy drinks. Because “sometimes agree” is ambiguous between genuinely occasional use and uncertainty about the question, the primary definition was deliberately restrictive; a sensitivity analysis using a broader definition that additionally counted “sometimes agree” as current use (441/725; 60.8%) is reported in Supplementary Table S3.

Knowledge and attitude scores. Composite scores were derived by summing the relevant five-point Likert items (knowledge: three items, Q15–Q17, range 3–15; attitudes: four items, Q19–Q21 and Q29, range 4–20). The knowledge items ask respondents to rate their own familiarity with supplements, their own knowledge of recommended doses, and their own awareness of risks; they do not test factual knowledge against a correct answer. The resulting variable is therefore reported throughout as self-rated knowledge, and is interpreted as perceived rather than verified competence. Scores were categorized by proportion of the maximum attainable score using the modified Bloom’s cut-off points of ≥80%, 60%–79%, and <60% of the maximum attainable score (35), which we label excellent/positive, moderate/neutral, and poor/negative, respectively. This convention is widely applied in nutrition and public-health KAP research, including in studies of supplement use in Arab populations (36, 37). A practice score was likewise derived among users but, for the reasons given in section 2.5, was not used as an explanatory variable.

Body mass index. BMI was calculated from self-reported height and weight as weight (kg) divided by height (m) squared and classified according to the World Health Organization adult categories: underweight <18.5 kg/m^2^, normal 18.5–24.9 kg/m^2^, overweight 25.0–29.9 kg/m^2^, and obese ≥30.0 kg/m^2^ (38). Before analysis, height and weight entries were audited: free-text entries were parsed to numeric values, heights entered in meters were converted to centimeters, and biologically implausible values (height outside 120–220 cm or weight outside 25–250 kg) were set to missing. BMI could not be computed for 37 respondents (5.1%) who did not disclose height, weight, or both.

### 2.5 Statistical analysis

All analyses were performed using IBM SPSS Statistics, version 26. Categorical variables were summarized as frequencies and percentages calculated on variable-specific available-case denominators, which are reported in every table, and compared between current users and non-users using the chi-square test; Fisher’s exact test was reserved for tables with an expected cell count below 5, but no such table arose (the smallest expected count in Table 1 was 10.0), so all reported *p*-values are from the chi-square test. No imputation was performed.

**Table 1:** Sociodemographic, lifestyle, and knowledge/attitude characteristics of respondents by current dietary supplement use (*N* = 725).

Characteristic	All respondents, *n* (%)	Current users, *n* (%)	Non-users, *n* (%)	*p*-value
Age (years) [*n* = 725]	725	291	434	0.147
18–30	366 (50.5)	162 (55.7)	204 (47.0)	
31–45	193 (26.6)	71 (24.4)	122 (28.1)	
46–60	131 (18.1)	46 (15.8)	85 (19.6)	
>60	35 (4.8)	12 (4.1)	23 (5.3)	
Sex [*n* = 725]	725	291	434	0.173
Male	493 (68.0)	189 (64.9)	304 (70.0)	
Female	232 (32.0)	102 (35.1)	130 (30.0)	
Marital status [*n* = 725]	725	291	434	0.015
Single	394 (54.3)	174 (59.8)	220 (50.7)	
Married	305 (42.1)	104 (35.7)	201 (46.3)	
Divorced	26 (3.6)	13 (4.5)	13 (3.0)	
Education [*n* = 725]	725	291	434	0.333
Bachelor’s degree	377 (52.0)	163 (56.0)	214 (49.3)	
High school	157 (21.7)	53 (18.2)	104 (24.0)	
Diploma	88 (12.1)	33 (11.3)	55 (12.7)	
Master’s degree	78 (10.8)	31 (10.7)	47 (10.8)	
PhD	25 (3.4)	11 (3.8)	14 (3.2)	
Region of residence [*n* = 725]	725	291	434	0.042
Riyadh Region	616 (85.0)	258 (88.7)	358 (82.5)	
Eastern Province	40 (5.5)	12 (4.1)	28 (6.5)	
Makkah	25 (3.4)	11 (3.8)	14 (3.2)	
Other regions	44 (6.1)	10 (3.4)	34 (7.8)	
Employment status [*n* = 719]	719	288	431	0.693
Employed	411 (57.2)	170 (59.0)	241 (55.9)	
Student	180 (25.0)	68 (23.6)	112 (26.0)	
Unemployed	128 (17.8)	50 (17.4)	78 (18.1)	
Monthly income (SAR) [*n* = 712]	712	287	425	0.082
>7,000	366 (51.4)	144 (50.2)	222 (52.2)	
3,000–7,000	141 (19.8)	68 (23.7)	73 (17.2)	
<3,000	205 (28.8)	75 (26.1)	130 (30.6)	
Chronic disease [*n* = 722]	722	290	432	0.795
No	597 (82.7)	238 (82.1)	359 (83.1)	
Yes	125 (17.3)	52 (17.9)	73 (16.9)	
Smoking status [*n* = 717]	717	287	430	0.645
No	529 (73.8)	211 (73.5)	318 (74.0)	
Yes	145 (20.2)	56 (19.5)	89 (20.7)	
Nicotine pouches only	43 (6.0)	20 (7.0)	23 (5.3)	
Following a special diet [*n* = 722]	722	291	431	<0.001
No	544 (75.3)	177 (60.8)	367 (85.2)	
Yes	178 (24.7)	114 (39.2)	64 (14.8)	
BMI category (kg/m^2^) [*n* = 688]	688	278	410	0.480
Normal (18.5–24.9)	253 (36.8)	110 (39.6)	143 (34.9)	
Overweight (25.0–29.9)	243 (35.3)	98 (35.3)	145 (35.4)	
Obese (≥30.0)	167 (24.3)	62 (22.3)	105 (25.6)	
Underweight (<18.5)	25 (3.6)	8 (2.9)	17 (4.1)	
Self-identified athlete [*n* = 725]	725	291	434	<0.001
Yes	348 (48.0)	180 (61.9)	168 (38.7)	
No	377 (52.0)	111 (38.1)	266 (61.3)	
Self-rated knowledge level [*n* = 725]	725	291	434	<0.001
Excellent	199 (27.4)	140 (48.1)	59 (13.6)	
Moderate	377 (52.0)	130 (44.7)	247 (56.9)	
Poor	149 (20.6)	21 (7.2)	128 (29.5)	
Attitude level [*n* = 724]	724	291	433	<0.001
Positive	298 (41.2)	178 (61.2)	120 (27.7)	
Neutral	345 (47.7)	108 (37.1)	237 (54.7)	
Negative	81 (11.2)	5 (1.7)	76 (17.6)	

Values are *n* (%). Percentages are column proportions calculated on variable-specific available-case denominators, which are shown in square brackets beside each characteristic and in the header row of each block; respondents who did not disclose a given item are excluded from that variable only and are not imputed or shown as a separate category. *p*-values are from the chi-square test comparing current users with non-users. Knowledge and attitude levels are the scored Likert categories used in the multivariable model (Table 2). BMI, body mass index; SAR, Saudi riyal. “Other regions” combines all administrative regions other than Riyadh, the Eastern Province, and Makkah.

#### 2.5.1 Model specification

Current dietary supplement use (yes = 1, no = 0) was entered as the dependent variable in a multivariable binary logistic regression model. The independent variables entered simultaneously were age band (reference 18–30 years), sex (reference male), marital status (reference married), education (reference bachelor’s degree), region of residence (dichotomized as Riyadh Region versus all other regions because of small numbers in individual provinces; reference other regions), monthly income (reference >7,000 SAR), chronic disease (reference no), smoking status (reference non-smoker), following a special diet (reference no), BMI category (reference normal), self-identified athlete status (reference no), self-rated knowledge level (reference excellent), and attitude level (reference positive). Results are expressed as adjusted odds ratios (aOR) with 95% confidence intervals (CI) and Wald statistics.

#### 2.5.2 Variable selection

The variables entered in the multivariable model were selected entirely *a priori*, before any modeling was undertaken, on conceptual and prior-evidence grounds. No data-driven selection procedure of any kind was used: candidate variables were not screened against the bivariable (chi-square) results shown in Table 1, no *p*-value threshold for entry or retention was applied, and no automated stepwise, forward, or backward algorithm was run. The inclusion criterion was therefore substantive rather than statistical. A variable was entered if it was either (i) a sociodemographic or lifestyle characteristic previously reported as a correlate of dietary supplement use, or as a plausible confounder of the association between knowledge or attitudes and use (age band, sex, marital status, education, region of residence, monthly income, chronic disease, smoking status, following a special diet, BMI category, and self-identified athlete status), or (ii) one of the two exposures of primary interest to the study objective (self-rated knowledge level and attitude level). The complete set of 13 variables so defined was entered simultaneously in a single block, and every variable was retained in the final model regardless of its statistical significance either in the unadjusted analysis or in the model itself, so that all reported estimates are mutually adjusted for the full set. Two consequences follow directly from this rule. First, age and sex were retained as *a priori* confounders on theoretical grounds even though neither was associated with use in the unadjusted analysis (age *p* = 0.147; sex *p* = 0.173); adjusted estimates for all four age bands and for sex are therefore reported in Table 2 and displayed in Figure 2 alongside every other variable in the model. Second, no variable was removed because of a non-significant univariable *p*-value. Only two of the characteristics shown in Table 1 were deliberately not entered, in both cases for a pre-specified structural reason rather than a statistical one. Employment status was omitted because its categories (employed, student, unemployed) are structurally confounded with age band and monthly income, both of which are in the model; in a sensitivity analysis adding it, employment status was not associated with use (student versus employed adjusted odds ratio 0.82, 95% CI 0.38–1.75; unemployed 1.20, 0.62–2.31), the maximum variance inflation factor rose from 3.07 to 3.58, and no other estimate changed materially. The practice category was omitted because practice items presuppose supplement use and are defined only among users, so entering practice as an explanatory variable for use would introduce circularity. The size of the *a priori* covariate set was constrained in advance by the number of available outcome events: with 270 events and 25 estimated parameters the model yields 10.8 events per variable, above the conventional minimum of 10 events per variable for stable logistic regression estimation (39).

**Table 2:** Multivariable binary logistic regression of factors associated with current dietary supplement use (*n* = 666 complete cases; 270 current users).

Variable	Adjusted OR	95% CI	Wald	*p*-value
Age (years) (ref. 18–30)				
31–45	0.63	0.32–1.21	1.92	0.166
46–60	0.72	0.32–1.62	0.63	0.427
>60	0.79	0.25–2.46	0.17	0.684
Sex (ref. male)				
Female	1.51	0.96–2.37	3.20	0.074
Marital status (ref. married)				
Single	1.18	0.63–2.24	0.27	0.604
Divorced	2.11	0.68–6.55	1.65	0.199
Education (ref. bachelor’s degree)				
High school	0.64	0.38–1.08	2.79	0.095
Diploma	0.49	0.26–0.94	4.66	0.031
Master’s degree	1.60	0.80–3.21	1.75	0.186
PhD	1.53	0.56–4.15	0.69	0.408
Region of residence (ref. other regions)				
Riyadh	0.97	0.56–1.67	0.02	0.901
Monthly income (SAR) (ref. >7,000)				
3,000–7,000	1.27	0.73–2.22	0.72	0.396
<3,000	0.69	0.39–1.19	1.78	0.183
Chronic disease (ref. no)				
Yes	1.90	1.09–3.33	5.07	0.024
Smoking status (ref. non-smoker)				
Yes	1.00	0.61–1.62	0.00	0.986
Nicotine pouches only	1.04	0.47–2.29	0.01	0.925
Following a special diet (ref. no)				
Yes	2.44	1.55–3.85	14.86	<0.001
BMI category (ref. normal)				
Overweight	1.08	0.69–1.70	0.12	0.733
Obese	1.15	0.67–1.95	0.25	0.615
Underweight	0.75	0.24–2.31	0.25	0.617
Self-identified athlete (ref. no)				
Yes	1.29	0.84–2.00	1.37	0.243
Self-rated knowledge level (ref. excellent)				
Moderate	0.34	0.22–0.53	23.32	<0.001
Poor	0.11	0.06–0.20	47.57	<0.001
Attitude level (ref. positive)				
Neutral	0.40	0.27–0.59	20.82	<0.001
Negative	0.07	0.03–0.20	25.12	<0.001

Dependent variable: current dietary supplement use (1 = current user, 0 = non-user). All listed variables were entered simultaneously in a single block and retained irrespective of statistical significance in unadjusted analysis; no stepwise or other automated selection was used. Adjusted OR, adjusted odds ratio; CI, confidence interval; ref., reference category; SAR, Saudi riyal; BMI, body mass index. Model diagnostics: McFadden pseudo-R^2^ = 0.25; model χ^2^ *p* < 0.001; Hosmer–Lemeshow *p* = 0.98; c-statistic 0.82 (95% CI 0.79–0.85); calibration plot in Supplementary Figure S1; maximum variance inflation factor 3.07; 10.8 events per estimated parameter. All performance measures are apparent (within-sample) estimates from the development sample and are optimistic to an unknown degree; no independent validation was undertaken. The practice category was not entered as an explanatory variable because practice items are defined only among users. Odds ratios overstate prevalence ratios when the outcome is common; corresponding adjusted prevalence ratios are given in Supplementary Table S4.

**Figure 2:**
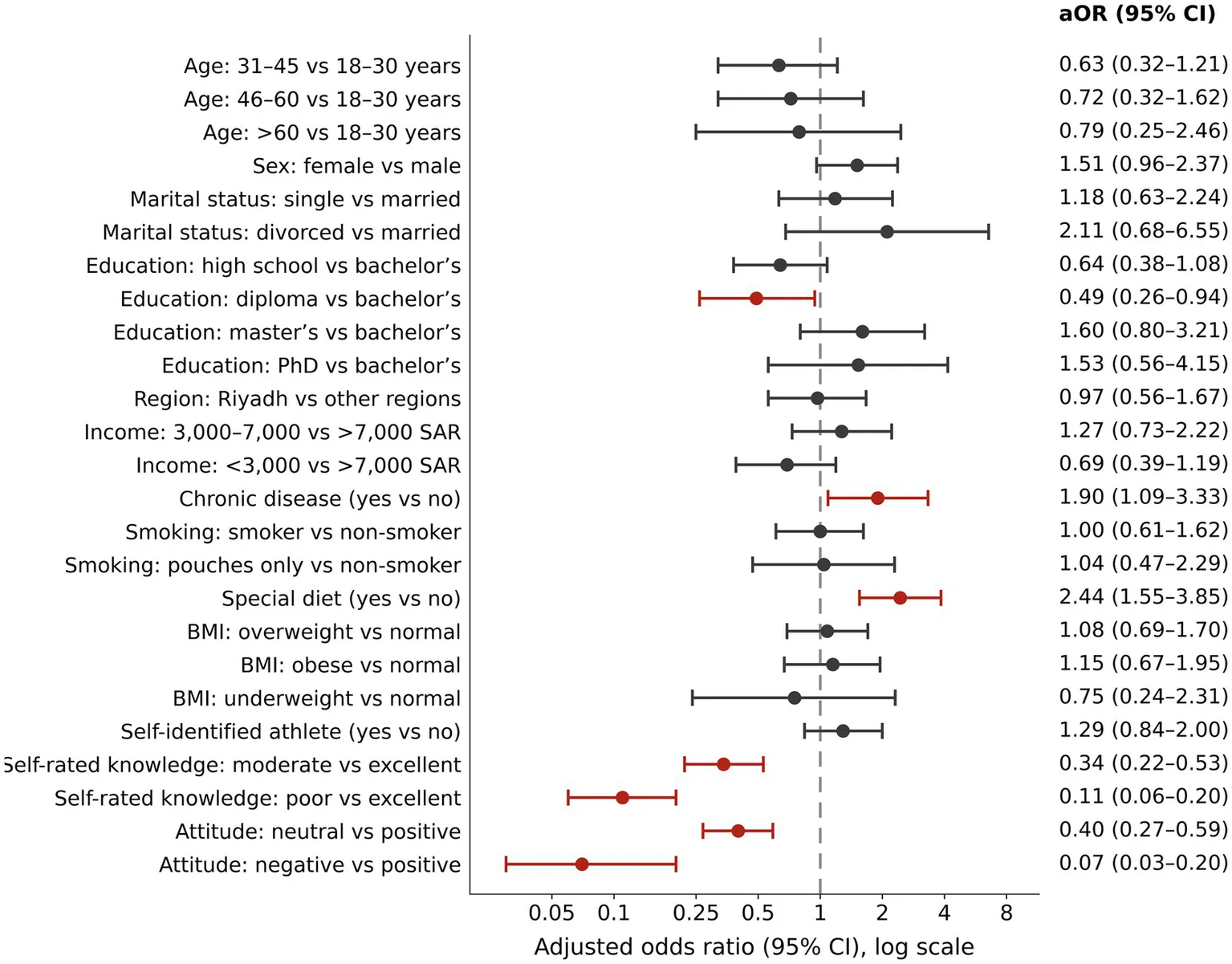
Adjusted odds ratios for every variable entered in the multivariable binary logistic regression model of current dietary supplement use (*n* = 666 complete cases; 270 current users). All 13 variables of the *a priori* covariate set are displayed, including age and the other variables that were not statistically significant; no variable was omitted either from the model or from this figure. Adjusted odds ratios are plotted with their 95% confidence intervals on a logarithmic scale; red markers indicate *p* < 0.05 and dark gray markers *p* ≥ 0.05, and the dashed vertical line marks an odds ratio of 1. Reference categories are: age, 18–30 years; sex, male; marital status, married; education, bachelor’s degree; region of residence, regions other than Riyadh; monthly income, >7,000 SAR; chronic disease, no; smoking status, non-smoker; special diet, no; BMI category, normal; self-identified athlete, no; self-rated knowledge, excellent; attitude, positive. “Smoking: pouches only” denotes respondents who used nicotine pouches but did not smoke. Because the outcome is current use and the most favorable category is the reference, ratios below 1 indicate that respondents in that category were less likely to be current users than those in the reference category. The corresponding numerical estimates, Wald statistics, and *p*-values are given in Table 2. BMI, body mass index; CI, confidence interval; SAR, Saudi riyal.

#### 2.5.3 Model performance

Multicollinearity was assessed using the variance inflation factor (VIF). Discrimination was assessed using the c-statistic (area under the receiver operating characteristic curve). Because a non-significant Hosmer–Lemeshow test is a weak and low-powered indicator of calibration, calibration was additionally assessed graphically: predicted probabilities were saved from the fitted model, grouped into deciles of predicted risk, and plotted against the observed proportion of users in each decile (40). This calibration plot is presented as Supplementary Figure S1. All model performance measures reported here are apparent (within-sample) estimates obtained in the development sample; because the model was not validated on an independent sample, they are optimistic to an unknown degree and are interpreted accordingly in section 4.

#### 2.5.4 Sensitivity analyses

Four pre-specified sensitivity analyses were conducted and are reported in Supplementary Table S3. First, because the outcome prevalence (40.1%) is high enough that odds ratios overstate prevalence ratios, adjusted prevalence ratios (aPR) were estimated using modified Poisson regression, that is, a generalized linear model with a Poisson distribution, log link, and a robust (Huber–White sandwich) covariance estimator (41), fitted over the identical covariate set; the full aPR model is reported as Supplementary Table S4. Second, the model was refitted with the continuous knowledge and attitude scores in place of the categorized variables. Third, the model was refitted excluding BMI and income, the two covariates responsible for most non-disclosure, in order to maximize the analyzed sample. Fourth, the model was refitted using the broader outcome definition described in section 2.4. In addition, respondents included in and excluded from the complete-case model were compared on all analyzed characteristics (Supplementary Table S5), and the implications of the missing-data mechanism are discussed in section 4. A two-sided *p* < 0.05 indicated statistical significance.

### 2.6 Ethics statement

The study was conducted in accordance with the ethical principles for medical research involving human participants set out in the World Medical Association Declaration of Helsinki, as revised by the 75th WMA General Assembly in Helsinki, Finland, in October 2024 (42). The protocol was approved by the Standing Committee on Research Ethics of King Saud University (approval number KSU-HE-26-0239; approval date 12 March 2026). Electronic informed consent was obtained from all participants before any study item was displayed; participation was voluntary and could be discontinued at any point without consequence; no identifying information was collected, and responses were analyzed anonymously in aggregate.

## 3 Results

### 3.1 Participant flow and characteristics

Of 748 submitted responses, 13 were removed because consent was declined and 2 because they were duplicate submissions, leaving 733 unique responses with a valid outcome; a further 8 were excluded because one or more key sociodemographic items were undisclosed, yielding 725 respondents for the descriptive analysis (Figure 1). The multivariable model was fitted on the 666 respondents (91.9%) with complete data on every covariate.

Of the 725 respondents, 291 (40.1%) were current DS users and 434 (59.9%) were non-users. The sample was predominantly young (50.5% aged 18–30 years), male (68.0%), educated to the bachelor’s level (52.0%), and residents of the Riyadh Region (85.0%); 48.0% self-identified as athletes. Full distributions, with variable-specific denominators, are given in Table 1. In unadjusted comparisons, current use was associated with marital status (*p* = 0.015), region of residence (*p* = 0.042), following a special diet (*p* < 0.001), self-identified athlete status (*p* < 0.001), self-rated knowledge level (*p* < 0.001), and attitude level (*p* < 0.001). Age, sex, education, employment, income, chronic disease, smoking, and BMI category were not associated with use in unadjusted analysis (all *p* > 0.05).

### 3.2 Knowledge and attitudes

Awareness of DS was near-universal (706/725; 97.4%), but self-rated practical competence was substantially lower (Supplementary Table S1). Only 32.0% agreed or strongly agreed that they knew the recommended doses, while 34.5% disagreed, making this the weakest item in the knowledge section. Self-rated general knowledge (47.6% agreeing) and awareness of risks (57.8% agreeing) fell between these extremes. The internet was by far the most frequently named single source of information (57.0%), while pharmacists were named by only 3.9%; dietitians (13.8%), friends or family (13.9%), and doctors (11.4%) occupied the middle of the distribution.

The composite knowledge score had a mean of 10.2 (SD 2.4; possible range 3–15) and the attitude score a mean of 14.8 (SD 3.1; possible range 4–20). Attitudes were generally favorable (Supplementary Table S2). Agreement was highest for supplement safety, with 82.9% agreeing that DS are safe when used correctly, and 60.7% considering them beneficial for general health. Views were more divided on universal necessity (35.2% agreeing, 30.3% disagreeing), and willingness to recommend supplements to others was predominantly conditional, with 42.9% selecting “sometimes agree.”

### 3.3 Practices among current users

Among the 291 current users, use was typically frequent and vitamin-based, and had usually been sustained for more than a month (Table 3). Daily use predominated (69.4%) and vitamins were the most commonly reported product (83.5%). Just over half (56.4%) had consulted a health professional before starting, and most reported no adverse effects (83.8%), while 8.6% reported adverse effects and a further 7.6% did not know whether they had experienced any. General health was the most frequently endorsed reason for use (70.4%).

**Table 3:** Dietary supplement practices among current users (*n* = 291).

Practice	*n*	%
Frequency of use [*n* = 291]		
Daily	202	69.4
Weekly	63	21.6
Monthly	15	5.2
Rarely	11	3.8
Type of supplement used^a^ [*n* = 291]		
Vitamins	243	83.5
Protein	136	46.7
Minerals	112	38.5
Herbal	47	16.2
Duration of use [*n* = 291]		
<1 month	49	16.8
1–6 months	132	45.4
>6 months	110	37.8
Consulted a health professional before use [*n* = 291]		
Yes	164	56.4
No	127	43.6
Reason for use^a^ [*n* = 291]		
General health	205	70.4
Sports/fitness	141	48.5
Nutritional deficiency	137	47.1
Advice/recommendation	42	14.4
Experienced side effects [*n* = 291]		
No	244	83.8
Yes	25	8.6
Do not know	22	7.6

Values are *n* and % of current users, calculated on the denominator shown in square brackets for each block.

a Multiple responses permitted; percentages may sum to more than 100%.

### 3.4 Sports-supplement practices among self-identified athletes

Of the 725 respondents, 348 (48.0%) self-identified as athletes engaged in regular training or competition. This subgroup is defined by athletic status alone and not by supplement use: the athlete module was administered to all 348 self-identified athletes irrespective of whether they were classified as current DS users, and all 348 responded to it. The sports-supplement items (Q32, Q33) asked which products the respondent uses without anchoring the question to the present, whereas the outcome item (Q22) asked specifically about current use. The two items are therefore not interchangeable, and 168 of the 348 athletes, all of whom reported at least one sports supplement type, were nonetheless classified as non-users under our restrictive outcome definition. Table 4 accordingly describes sports-supplement practices reported by self-identified athletes, not the current-use behavior that defines the outcome. Athletes were more likely than non-athletes to be current DS users (180/348, 51.7% versus 111/377, 29.4%; *p* < 0.001), and the 180 athlete users accounted for 61.9% of the 291 current users in the sample. The percentages in Table 4 are therefore calculated on a denominator of 348 self-identified athletes, not on the 291 current users.

**Table 4:** Sports-supplement practices among self-identified athletes (*n* = 348).

Practice	*n*	%
Sports supplements used^a^ [*n* = 348]		
Protein	210	60.3
Vitamins	196	56.3
Creatine	153	44.0
Sports drinks	83	23.9
BCAA	52	14.9
Electrolytes	36	10.3
Goal of sports-supplement use^a^ [*n* = 348]		
Improve performance	238	68.4
Maintain health	214	61.5
Increase energy	192	55.2
Accelerate recovery	151	43.4
Reads ingredients before use [*n* = 348]		
Strongly agree	134	38.5
Agree	99	28.4
Sometimes agree	79	22.7
Disagree	25	7.2
Strongly disagree	11	3.2
Checks products for banned substances [*n* = 348]		
Strongly agree	161	46.3
Agree	67	19.3
Sometimes agree	52	14.9
Disagree	40	11.5
Strongly disagree	28	8.0
Main guidance source [*n* = 348]		
Internet	127	36.5
Doctor	74	21.3
Teammates/friends	54	15.5
Dietitian	49	14.1
Coach	44	12.6
Usual purchasing channel [*n* = 348]		
Pharmacy	141	40.5
Online	96	27.6
Sports store	95	27.3
Club store	16	4.6
Blood testing before or during supplement use [*n* = 348]		
No	159	45.7
Yes, before use	131	37.6
Yes, during use	47	13.5
Not applicable	11	3.2
Experienced supplement-related side effects [*n* = 348]		
No	271	77.9
Yes	23	6.6
Do not know	54	15.5

Values are *n* and % of self-identified athletes, calculated on the denominator shown in square brackets for each block. The denominator is all 348 respondents who self-identified as athletes engaged in regular training or competition, irrespective of whether they were classified as current dietary supplement users (180 of the 348 athletes, 51.7%, were current users). BCAA, branched-chain amino acids. “Not applicable” for blood testing denotes athletes who indicated the item did not apply to them.

a Multiple responses permitted; percentages may sum to more than 100%.

Within this subgroup, protein (60.3%), vitamins (56.3%), and creatine (44.0%) were the most frequently reported sports supplements, used principally to improve performance (68.4%) and to maintain health (61.5%) (Table 4). Two-thirds reported reading ingredient labels before use (67.0% agreeing or strongly agreeing) and checking products for banned substances (65.5%), although 19.5% disagreed with the latter. The internet again led as a guidance source (36.5%), ahead of doctors (21.3%), teammates or friends (15.5%), dietitians (14.1%), and coaches (12.6%). Pharmacies were the most common purchasing channel (40.5%), followed by online retailers (27.6%) and sports stores (27.3%). Biochemical monitoring was limited: 45.7% had never undergone blood testing in relation to supplement use, whereas 37.6% had been tested before starting and 13.5% during use. Supplement-related side effects were reported by 6.6%, denied by 77.9%, and a further 15.5% were unsure whether they had experienced any.

### 3.5 Factors independently associated with current use

In the multivariable model (Table 2; *n* = 666 complete cases, 270 users), seven coefficients reached statistical significance. Following a special diet was associated with more than a doubling of the odds of current use (aOR 2.44, 95% CI 1.55–3.85, *p* < 0.001). Graded inverse associations were observed for both KAP domains: relative to excellent self-rated knowledge, the odds of current use were lower for moderate (aOR 0.34, 95% CI 0.22–0.53) and poor self-rated knowledge (aOR 0.11, 95% CI 0.06–0.20), and relative to positive attitudes, they were lower for neutral (aOR 0.40, 95% CI 0.27–0.59) and negative attitudes (aOR 0.07, 95% CI 0.03–0.20) (all *p* < 0.001). Because current use is the outcome and the most favorable category is the reference, these ratios below unity indicate that respondents with less favorable self-rated knowledge or attitudes were less likely to be current users; equivalently, current users were more likely than non-users to rate their own knowledge highly and to endorse supplements. In the complete-case sample, the negative-attitude stratum contained only 5 users among 73 respondents (81 respondents in the full descriptive sample; Table 1), so that estimate is imprecise. Chronic disease (aOR 1.90, 95% CI 1.09–3.33, *p* = 0.024) and diploma-level relative to bachelor-level education (aOR 0.49, 95% CI 0.26–0.94, *p* = 0.031) each showed a nominally significant but imprecise association with use, with a confidence interval close to unity. Marital status and athlete status, both associated with use in unadjusted analysis, were not independently associated after adjustment (single versus married aOR 1.18, 95% CI 0.63–2.24; athlete aOR 1.29, 95% CI 0.84–2.00). Age, sex, region, income, smoking, and BMI category were not independently associated with use. The adjusted associations for all 25 estimated coefficients, including those for age, are displayed as a forest plot in Figure 2.

The model discriminated well (c-statistic 0.82, 95% CI 0.79–0.85; McFadden pseudo-R^2^ = 0.25; model χ^2^ *p* < 0.001) and showed no problematic multicollinearity (maximum VIF 3.07). The Hosmer–Lemeshow test was non-significant (*p* = 0.98), which, for the reason given in section 2.5, is weak evidence in itself; the calibration plot, which is the more informative assessment, lay close to the diagonal across the full range of predicted risk (Supplementary Figure S1). We do not report a calibration slope or calibration-in-the-large, because in the sample in which a logistic model is fitted, these take the values 1 and 0 by construction and carry no information. These are apparent, within-sample estimates obtained in the development sample. Because the model was not validated in an independent sample, they are optimistic to an unknown degree; the c-statistic in particular should be read as an upper bound, and we make no claim about how the model would perform outside this sample.

All four sensitivity analyses supported the primary model (Supplementary Tables S3, S4). Modified Poisson regression yielded adjusted prevalence ratios in the same directions but, as expected for a common outcome, closer to unity (special diet aPR 1.39, 95% CI 1.16–1.67; poor self-rated knowledge aPR 0.30, 95% CI 0.20–0.45; negative attitude aPR 0.18, 95% CI 0.08–0.42); the only material difference in inference was for sex, which reached nominal significance on the prevalence-ratio scale (female aPR 1.23, 95% CI 1.01–1.48) but not on the odds scale. Using continuous rather than categorized scores, each additional point of knowledge (aOR 1.38, 95% CI 1.25–1.52) and of attitude (aOR 1.32, 95% CI 1.23–1.43) was associated with higher odds of use, corresponding to adjusted odds ratios per standard deviation of 2.13 (95% CI 1.70–2.68) for knowledge and 2.39 (1.88–3.03) for attitude. Refitting the model without BMI and income (*n* = 712) and refitting it with the broader outcome definition (*n* = 666; 405 classified as users) left the special-diet, knowledge, and attitude associations essentially unchanged. Respondents excluded from the complete-case model (*n* = 59) were more often female (50.8% versus 30.3%, *p* = 0.002) and differed in employment status (*p* = 0.008) but did not differ in current DS use (35.6% versus 40.5%, *p* = 0.55), self-rated knowledge (*p* = 0.07), or attitude level (*p* = 0.78) (Supplementary Table S5).

## 4 Discussion

In this cross-sectional online survey of 725 adults residing in Saudi Arabia, 2 in 5 reported current DS use against a backdrop of near-universal awareness, limited self-rated competence in dosing, and heavy reliance on the internet rather than health professionals as an information source. These patterns align with Saudi and regional KAP studies and reinforce a consistent theme: supplement use is common amid near-universal awareness but limited self-rated competence in the practical knowledge needed to use it safely (8, 14–16, 29, 30). The current-use prevalence of 40.1% is lower than the 58%–71% reported in Saudi surveys that captured any or past-year use (8, 31), which is consistent with our deliberately restrictive definition of current use; under the broader definition that also counted occasional users, prevalence rose to 60.8%, within the range those studies report.

The dissociation between awareness and self-rated knowledge of dosing is the most actionable descriptive finding. Incorrect dosing is a principal driver of supplement-related harm (19, 43), so the observation that only about a third of respondents were confident about correct doses, while more than four in five regarded supplements as safe when used correctly, is worth attention. We are careful, however, not to over-read this pattern in two respects. First, confidence about dosing is a self-appraisal and not a demonstration of competence: our instrument did not test factual knowledge, so a low proportion agreeing that they know correct doses indicates low perceived competence, which may over- or understate actual competence. Second, agreeing that supplements are safe “when used correctly” is a conditional statement that is, in itself, defensible rather than evidence of safety optimism; it becomes a concern only when it coexists with low confidence about what correct use entails, which is the specific configuration observed here. The appropriate reading is therefore a mismatch between endorsement and self-assessed procedural knowledge, rather than a demonstrated overestimate of safety.

The role of health professionals also requires a distinction of the descriptive data made available. Pharmacists were selected as the single main source of general information by only 3.9% of respondents, but 56.4% of current users reported consulting a health professional before starting a supplement. These are different behaviors measured by different items: the first identifies the channel through which respondents ordinarily learn about supplements, the second whether a specific consultation preceded use. Professional involvement in the decision to use supplements is therefore substantially more common than the information-source item alone would suggest, and it is the routine informational channel, not the point-of-decision consultation, that is dominated by unverified online content. The contrast with settings such as Dubai, where supplements are more often taken on the advice of pharmacists and other health professionals (9, 30), remains instructive, and the low salience of pharmacists as an everyday information source is notable given their position to detect drug–supplement interactions.

Turning to the modeled associations, following a special diet, self-rated knowledge level, and attitude level were the factors most clearly associated with current use after adjustment, whereas marital status and athlete status did not persist. Supplement use was more common among respondents following a special diet, a clustering also reported in Saudi and US data (16, 31, 44). Because the diet type was not recorded, however, this cannot be attributed to health-attentiveness rather than to medically indicated dietary management, two situations with different counseling implications. In contrast to earlier Saudi analyses, which identified mainly demographic correlates such as sex and education (31), the dominant independent correlates here were self-rated knowledge and attitude. We deliberately refrain from converting this observation into a causal or interventional claim. The design is cross-sectional and cannot establish temporal order; the most plausible interpretation is bidirectional, and reverse causation is at least as likely as the forward direction, since people who take supplements are repeatedly exposed to product information and have an obvious incentive to view supplements favorably. Knowledge and attitudes should therefore be read as correlates that travel with use, not as demonstrated modifiable causes of it, and the graded pattern across categories, replicated on the prevalence-ratio scale and with continuous scores, should be understood as a consistent association rather than a dose–response effect. Chronic disease reached significance only after adjustment, with a confidence interval close to unity, and should be regarded as hypothesis-generating. The isolated diploma-level education contrast should be read the same way, and more skeptically still: it showed no gradient across the five education categories, its confidence interval abuts unity, and it is one of seven nominally significant coefficients among 25 estimated without adjustment for multiplicity, so chance is the most parsimonious explanation. We did not enter practice as an explanatory variable, because practice is measured only among users and would create circularity; this differs from some prior analyses and should be borne in mind in comparisons.

The athlete subgroup adds practical nuance. Self-reported anti-doping awareness appeared relatively high, with about two-thirds of athletes reading labels and checking for banned substances, a more favorable picture than among Lebanese athletes (25) and broadly comparable to Saudi and Gulf fitness-center and professional-athlete populations (45–48). Supplement use is consistently higher in athletes than in the general population (26, 49–51), and the two products reported by our athletes that carry a robust evidence base, protein (60.3%) and creatine (44.0%), were among the most commonly used, although vitamins (56.3%), for which an ergogenic rationale is weaker, were reported nearly as often (52–54). Nevertheless, expert bodies recommend a full nutritional assessment before supplement use (27), because sports supplements are frequently contaminated or adulterated with prohibited substances and are a recognized cause of inadvertent anti-doping rule violations (28, 55, 56); athletes are therefore advised to follow structured decision frameworks and to use batch-tested products (43, 57, 58). That internet-led guidance outranked dietitians and coaches, that more than a quarter of athletes bought supplements online, that almost half had never had blood testing in relation to supplement use, and that about one in six was unsure whether they had experienced side effects, together identify concrete targets for sports-nutrition education and monitoring.

### 4.1 The Saudi regulatory context and feasible national responses

Interpreting these findings requires the specific Saudi regulatory setting rather than general international commentary on “unregulated” supplements. Supplements are not unregulated in Saudi Arabia. The Saudi Food and Drug Authority (SFDA) classifies orally consumed products containing vitamins, minerals, amino acids, herbal extracts, and similar ingredients as food supplements, distinguishes them from health products presented in pharmaceutical dosage forms, and classifies any product presented as treating, preventing, or diagnosing disease as a medicine, regardless of its ingredients (59). Marketing is likewise governed: SFDA rules permit function and general well-being claims but prohibit claims to treat, cure, or prevent disease, and prohibit “clinically proven,” “no side effects”, and “100% safe” wording on supplements (60), and advertisements for products under SFDA supervision require prior approval (61). Post-marketing surveillance also exists and reaches supplement-type products: SFDA has screened registered herbal products for herb–drug interaction signals and added confirmed interactions to product information (62), while national survey data indicate that supplement- and herbal-related adverse events do reach community pharmacists (63).

The gap our data identify is therefore not an absence of regulation but a distance between the regulated channel and the informational one. Most respondents (57.0%) named the internet as their main source of information about supplements, more than a quarter of athletes bought their products online, and pharmacists, who are positioned at the regulated point of sale, are covered by the pharmacovigilance system, and can detect drug–supplement interactions, were named as the main information source by fewer than 1 in 25 respondents. Several responses follow that are feasible within existing Saudi structures rather than requiring new legislation: professionally curated, Arabic-language digital supplement information that can compete on the platforms where respondents actually look; extension of the existing advertising pre-approval mechanism to the social-media and influencer marketing that carries much supplement promotion; a structured supplement-counseling role for community pharmacists at the point of dispensing, supported by dietitian referral for individuals following special diets or managing chronic conditions; and public-facing promotion of the SFDA adverse-event reporting route, given that one in thirteen users and about one in six athletes could not say whether they had experienced side effects, and that 45.7% of athletes had never had blood testing in relation to their supplement use. For athletes specifically, alignment with anti-doping guidance on batch-tested products and structured decision frameworks (27, 57) is a concrete extension of the same principle.

### 4.2 Strengths and limitations

The principal limitation is external validity, and it constrains every estimate reported here. The sample was recruited by non-probability convenience sampling and is not representative of the Saudi adult population: respondents were predominantly male (68.0%), young (50.5% aged 18–30 years), and heavily concentrated in the Riyadh Region (85.0%), and self-identified athletes comprised 48.0%, a proportion far above any plausible population value and a clear sign that recruitment reached fitness-oriented communities. Prevalence estimates in particular should not be read as national figures; the 40.1% current-use prevalence describes this sample only, and the athlete over-representation is likely to inflate supplement-related estimates. The predominance of male and younger respondents also means the findings may not capture supplementation among older adults, in whom use is particularly common (20, 64), or among women. We have accordingly stated the sample composition in the title and abstract, and we make no claim that the findings generalize beyond this sample or to other national settings.

Second, the cross-sectional design precludes causal inference and cannot establish whether knowledge and attitudes precede or follow supplement use, as discussed above. Third, all data are self-reported and subject to social-desirability and recall bias; anonymity mitigates but does not remove this. Because the survey was anonymous and required no sign-in, age, residence, and consent were self-declared and could not be independently verified, and repeat submission could only be screened for *post hoc*. Fourth, the outcome rests on a single Likert item without a defined recall window, and does not separate prescribed from self-initiated supplement use; the sensitivity analysis using the broader definition addresses part, but not all, of this uncertainty. Fifth, the three-item knowledge scale showed only modest internal consistency (α = 0.60) and measures self-rated rather than verified knowledge, so the knowledge findings should be interpreted as perceived competence. Relatedly, the knowledge and attitude items and the outcome item were all self-reported on the same instrument at the same sitting, so the associations between them share method variance and the reported discrimination (c-statistic 0.82) is inflated by that shared source; the model is offered as a description of association within this sample, not as a prediction tool. Sixth, chronic disease and special diet were captured as single yes/no items, so neither the conditions nor the diet types can be described. Nationality was not recorded either; eligibility was defined by residence, and because a substantial share of the adult population of Saudi Arabia is non-national, the sample cannot be characterized on that axis and should be read as describing adults resident in the country rather than Saudi nationals. Seventh, the primary model used complete cases (*n* = 666; 8.1% excluded), with attrition reflecting elective non-disclosure of optional items: height or weight (*n* = 37), monthly income (*n* = 13), smoking status (*n* = 8), chronic disease (*n* = 3), special diet (*n* = 3), and one attitude item (*n* = 1), overlapping to give 59 excluded respondents. Complete-case analysis is unbiased under a missing-completely-at-random mechanism and, for the covariates concerned, under missingness that depends only on observed covariates; excluded respondents were more often female and differed in employment status, so the missing-completely-at-random assumption is unlikely to hold strictly, but they did not differ in the outcome or in the two main explanatory variables, and the sensitivity analysis omitting BMI and income reproduced the findings on a larger sample. Residual bias from this source is therefore likely to be small, though it cannot be excluded. Finally, all measures of model performance are apparent estimates from the development sample; no independent validation was undertaken, so the reported discrimination is an upper bound, and the model should not be treated as a validated prediction tool.

Set against these limitations, the study has several strengths: a sample well above the calculated minimum; an instrument adapted from previously published KAP tools with transparent reliability reporting, including of the scale that performed least well; an *a priori*, fully adjusted model with no data-driven variable selection; formal discrimination and calibration diagnostics, including a calibration plot, rather than point estimates alone; four pre-specified sensitivity analyses, including on the prevalence-ratio scale and under an alternative outcome definition; and complete item-level reporting of the knowledge and attitude distributions in the Supplementary material.

## 5 Conclusion

In this predominantly young, male, Riyadh-based convenience sample of 725 adults residing in Saudi Arabia, current DS use was common and attitudinally endorsed, but self-rated competence in dosing and reliance on professional rather than online information channels were limited. Self-rated knowledge and attitudes were associated with current use more strongly than any demographic characteristic; because the design is cross-sectional, these associations cannot be interpreted as causal, and their direction requires longitudinal confirmation before health-literacy interventions can be justified on the basis of these data. What the descriptive findings point to, independently of the modeled associations, is a shift in the object of counseling: the aim should be safer, evidence-based, and clinically appropriate supplement decision-making (knowing when a supplement is indicated, at what dose, from a regulated source, and with what interaction risk) rather than greater supplement knowledge or greater supplement use as ends in themselves. Prospective, probability-based studies with objective knowledge assessment are needed to establish whether improving these competencies changes behavior.

## Data Availability

The original contributions presented in the study are included in the article/Supplementary material, further inquiries can be directed to the corresponding author.

## Supplementary material

SUPPLEMENTARY FIGURE S1 Calibration plot for the multivariable logistic regression model, showing the observed proportion of current dietary supplement users against the predicted probability of use, by decile of predicted risk.
