# Supplementary material for: Knowledge and attitudes as correlates of dietary supplement use among adults in Saudi Arabia: a cross-sectional online survey of a predominantly Riyadh-based convenience sample
Source: Front Public Health. 2026 Sep 29;14:1929339. doi: 10.3389/fpubh.2026.1929339 (PMC13658588; doi:10.3389/fpubh.2026.1929339)
Supplement: Supplementary file 1 [file Data_Sheet_1.pdf]

Supplementary Data Sheet 1. Study questionnaire (Arabic, with English translation)

Knowledge and attitudes as correlates of dietary supplement use among adults in Saudi Arabia: a cross-sectional online survey of a predominantly Riyadh-based convenience sample

Informed consent

You are invited to take part in a research study on knowledge, attitudes, and practices regarding dietary supplement use among adults in Saudi Arabia. Participation is voluntary and anonymous; you may withdraw at any time without consequence. No personally identifying information is used in the analysis, and responses are reported only in aggregate. By selecting "Yes, I agree to participate," you confirm that you are 18 years or older, reside in Saudi Arabia, and consent to participate. The study was approved by the Standing Committee on Research Ethics of King Saud University (approval number KSU-HE-26-0239; approval date 12 March 2026).

Q1. Do you agree to participate in this study?

هل توافق على المشاركة في هذه الدراسة؟

Response options: Yes, I agree to participate / No, I do not wish to participate

Section A. Sociodemographic and lifestyle characteristics

Q2. Age

العمر

Response options: 18–30 / 31–45 / 46–60 / More than 60 (years)

Q3. Sex

الجنس

Response options: Male / Female

Q4. Marital status

الحالة الاجتماعية

Response options: Single / Married / Divorced

Q5. Education level

المستوى التعليمي

Response options: High school / Diploma / Bachelor’s / Master’s / PhD

Q6. Region of residence

منطقة السكن

Response options: Riyadh Region / Makkah / Eastern Province / other administrative regions of Saudi Arabia

Q7. Employment status

الحالة الوظيفية

Response options: Employed / Student / Unemployed

Q8. Monthly income (Saudi riyal)

الدخل الشهري (ريال سعودي)

Response options: Less than 3000 / 3000–7000 / More than 7000

Q9. Do you have a chronic disease?

هل تعاني من مرض مزمن؟

Response options: Yes / No

Q10. Are you a smoker?

هل أنت مدخن؟

Response options: No / Yes / Uses nicotine pouches (e.g., "Dessert")

Q11. Do you follow a special diet?

هل تتبع نظامًا غذائيًا خاصًا؟

Response options: Yes / No

Q12. Height (cm)

الطول (بالسنتيمتر)

Response options: Free numeric entry

Q13. Weight (kg)

الوزن (كيلوجرام)

Response options: Free numeric entry

Section B. Knowledge

Q14. Have you heard of dietary supplements before?

هل سمعت عن المكملات الغذائية من قبل؟

Response options: Yes / No

Q15. How would you rate your knowledge of dietary supplements?

ما مستوى معرفتك بالمكملات الغذائية؟

Response options: Strongly agree / Agree / Sometimes agree / Disagree / Strongly disagree

Q16. Do you know the recommended doses for dietary supplements?

هل تعرف الجرعات الموصى بها للمكملات الغذائية؟

Response options: Strongly agree / Agree / Sometimes agree / Disagree / Strongly disagree

Q17. Are you aware of the risks or side effects of supplements?

هل تعلم بوجود مخاطر أو آثار جانبية للمكملات؟

Response options: Strongly agree / Agree / Sometimes agree / Disagree / Strongly disagree

Q18. What is your main source of information about supplements?

ما هو مصدر معلوماتك الرئيسي عن المكملات؟

Response options: Internet / Doctor / Pharmacist / Friends / Dietitian

Section C. Attitudes

Q19. I believe dietary supplements are beneficial for general health

أعتقد أن المكملات الغذائية مفيدة للصحة العامة

Response options: Strongly agree / Agree / Sometimes agree / Disagree / Strongly disagree

Q20. I believe dietary supplements are safe when used correctly

أعتقد أن المكملات الغذائية آمنة عند استخدامها بشكل صحيح

Response options: Strongly agree / Agree / Sometimes agree / Disagree / Strongly disagree

Q21. I consider dietary supplements necessary for everyone

أرى أن المكملات الغذائية ضرورية لجميع الأشخاص

Response options: Strongly agree / Agree / Sometimes agree / Disagree / Strongly disagree

Section D. Practices and current use

Q22. Do you currently use dietary supplements?

هل تستخدم مكملات غذائية حاليًا؟

Response options: Strongly agree / Agree / Sometimes agree / Disagree / Strongly disagree

Q23. Frequency of supplement use

معدل استخدام المكملات

Response options: Daily / Weekly / Monthly / Rarely

Q24. Type of supplement used (select all that apply)

نوع المكمل الغذائي المستخدم)يمكن اختيار أكثر من جواب (

Response options: Vitamins / Protein / Minerals / Herbal products / Others

Q25. Duration of use

مدة الاستخدام

Response options: Less than 1 month / 1–6 months / More than 6 months

Q26. Did you consult a health professional before use?

هل استشرت مختصًا صحيًا قبل الاستخدام؟

Response options: Yes / No

Q27. Main reason for use (select all that apply)

السبب الرئيسي للاستخدام)يمكن اختيار أكثر من جواب (

Response options: General health / Sports or fitness / Nutritional deficiency / Others

Q28. Have you experienced any side effects?

هل ظهرت لديك آثار جانبية؟

Response options: Yes / No / Do not know

Q29. Would you recommend supplement use to others?

هل تنصح الآخرين باستخدام المكملات الغذائية؟

Response options: Strongly agree / Agree / Sometimes agree / Disagree / Strongly disagree

Section E. Sports-supplement module (self-identified athletes)

Q30. Are you an athlete who trains regularly or competes?

هل أنت رياضي وتمارس نشاطًا رياضيًا منتظمًا أو تشارك في منافسات؟

Response options: Yes / No

Q31. Do you use dietary supplements for sporting purposes?

هل تستخدم مكملات غذائية لأغراض رياضية؟

Response options: Strongly agree / Agree / Sometimes agree / Disagree / Strongly disagree

Q32. Types of sports supplements used (select all that apply)

ما أنواع المكملات الرياضية التي تستخدمها؟)اختر كل ما ينطبق(

Response options: e.g., Protein / Creatine / Vitamins / Amino acids / Others

Q33. Main goal of using sports supplements (select all that apply)

ما الهدف الرئيسي من استخدام المكملات الرياضية؟)يمكن اختيار أكثر من جواب(

Response options: Improve performance / Maintain health / Build muscle / Others

Q34. Do you use supplements to improve competitive performance?

هل تستخدم المكملات لتحسين الأداء التنافسي؟

Response options: Strongly agree / Agree / Sometimes agree / Disagree / Strongly disagree

Q35. Do you read the ingredients or nutrition facts before use?

هل تقرأ المكونات أو القيم الغذائية قبل الاستخدام؟

Response options: Strongly agree / Agree / Sometimes agree / Disagree / Strongly disagree

Q36. Who guides you in using supplements?

من يوجّهك لاستخدام المكملات؟

Response options: Dietitian / Doctor / Internet / Peers / Coach

Q37. Have you experienced any side effects from supplements?

هل تعرضت لأي آثار جانبية بسبب المكملات؟

Response options: Yes / No / Do not know

Q38. If yes, what symptoms?

ما هي الأعراض؟

Response options: Free text / multiple options

Q39. Do you check that supplements are free of banned substances (doping agents)?

هل تتحقق من خلو المكملات من المواد المحظورة)المنشطات(؟

Response options: Strongly agree / Agree / Sometimes agree / Disagree / Strongly disagree

Q40. Where do you usually buy your sports supplements?

من أين تشتري مكملاتك الرياضية غالبًا؟

Response options: Online / Pharmacy / Sports store / Club store

Q41. Did you undergo blood tests before or during supplement use?

هل أجريت فحوصات دم قبل أو أثناء استخدام المكملات؟

Response options: Yes, before use / Yes, during use / No / Not applicable
